# Dynamic evolution of the mTHF gene family associated with primary metabolism across life

**DOI:** 10.1186/s12864-024-10159-8

**Published:** 2024-05-01

**Authors:** Adam M. Rork, Arthi S. Bala, Tanya Renner

**Affiliations:** 1 Department of Entomology, The Pennsylvania State University, University Park, Pennsylvania, 16802 USA; 2 Department of Entomology, Purdue University, West Lafayette, Indiana, 47907 USA; 3 Department of Biochemistry and Molecular & Cellular Biology, Georgetown University, Washington, DC 20007 USA; 4 School of Medicine, Georgetown University, Washington, DC 20007 USA

**Keywords:** Archaea, Eukarya, Bacteria, Evolution, Folate cycle, Gene family, One-carbon metabolism

## Abstract

**Background:**

The folate cycle of one-carbon (C1) metabolism, which plays a central role in the biosynthesis of nucleotides and amino acids, demonstrates the significance of metabolic adaptation. We investigated the evolutionary history of the methylenetetrahydrofolate dehydrogenase (mTHF) gene family, one of the main drivers of the folate cycle, across life.

**Results:**

Through comparative genomic and phylogenetic analyses, we found that several lineages of Archaea lacked domains vital for folate cycle function such as the mTHF catalytic and NAD(P)-binding domains of *FolD*. Within eukaryotes, the mTHF gene family diversified rapidly. For example, several duplications have been observed in lineages including the Amoebozoa, Opisthokonta, and Viridiplantae. In a common ancestor of Opisthokonta, *FolD* and *FTHFS* underwent fusion giving rise to the gene *MTHFD1*, possessing the domains of both genes.

**Conclusions:**

Our evolutionary reconstruction of the mTHF gene family associated with a primary metabolic pathway reveals dynamic evolution, including gene birth-and-death, gene fusion, and potential horizontal gene transfer events and/or amino acid convergence.

## Background

Metabolism is a defining feature of life on Earth, allowing organisms to respond to environmental stimuli, grow, reproduce, maintain homeostasis, and regenerate cellular energy currency. From its ubiquity arises substantial diversity. Indeed, not only is metabolism diverse at the interspecific level, but at the intraspecific and individual levels, too. For example, insects are rather unique relative to other flighted animals in that their primary source of energy for fueling flight is the amino acid L-proline, which is ultimately converted to 2-oxoglutarate [1]. This is distinct from energy metabolism observed in the muscle cells of vertebrates, or even insects for non-flight purposes, where sugars such as L-glucose (stored as glycogen) are used to regenerate ATP [2]. A classic example of intraspecific variation can be seen in the ability of humans to metabolize lactose. While most humans express lactase as infants, its persistence wanes into adulthood for many, leading to lactose intolerance [3]. Certain alleles, however, have been shown to lead to the persistence of lactase expression post-infancy, allowing for the consumption of lactose in adults without medication or gastrointestinal irritation [4].

There are also many examples of distantly related taxa converging upon similar metabolic traits as well, such as the tendency for freeze-tolerant animals to sequester high concentrations of monosaccharides and polyols in their tissues to survive sub-zero temperatures. For example, the wasp *Polistes exclamans*, the frog *Lithobates sylvaticus*, and the turtle *Chrysemys picta* all utilize sugars (e.g. D-glucose and D-fructose) as cryoprotectants [5–7]. Features of metabolism are also regularly co-opted; that is, they evolve novel functions other than that which they initially evolved for. An excellent example of this involves the folate cycle of one-carbon (C1) metabolism. Throughout life, this pathway generates intermediates in the biosynthesis of purines and thymidine [8]. In certain insects, however, such as the ant *Camponotus pennsylvanicus* or the ground beetle *Harpalus pensylvanicus*, this cycle is likely utilized to biosynthesize relatively large quantities of formic acid for use in self-defense [9, 10]. Typically, the overproduction of formate via this cycle is indicative of cellular dysfunction such as oxidative cancers, but in these insects and likely others, it is a critical means of self-defense and survival [11].

**Fig. 1:**
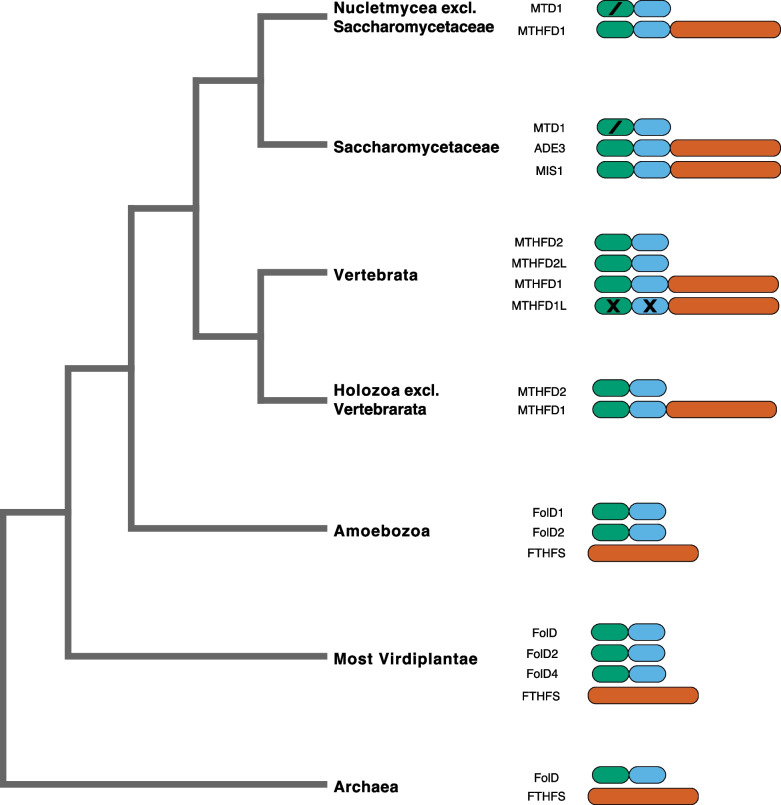
mTHF gene family architecture across life. Representation of mTHF gene family architecture across life. The mTHF gene family is diverse, especially in Eukarya where there has been gene birth and death, neofunctionalization, and gene fusion. The domains of each gene are represented in green, blue, and orange blocks. The green block illustrates the THF/DHG/CYC catalytic domain, the black block illustrates the NAD(P)^+^ binding domain, and the orange block illustrates the formyltetrahydrofolate synthetase domain. The block size represents the average length of the domains relative to one another. The single black slash represents domains which have undergone neofunctionalization function while two black slashes represent loss of function

Across life, the generation and utilization of C1 units by cells is necessary for the biosynthesis and regeneration of key metabolites such as nucleobases, amino acids, fatty acids, coenzymes, choline, etc. [8, 11–14]. While C1 units that feed into these processes may be derived from a variety of sources, amongst the most common are L-serine, L-glycine, and formate via the folate cycle of C1 metabolism [8, 12, 15]. The core of the folate cycle in vertebrates is composed of six nuclear -encoded enzymes, SHMT1, SHMT2, MTHFD1, MTHFD1L, MTHFD2, and MTHFD2L. SHMT1 and MTHFD1 are cytosolic, while SHMT2, MTHFD1L, MTHFD2, and MTHFD2L are localized to the mitochondria [8, 12, 16]. SHMT1 and SHMT2 are serine hydroxymethyltransferases (IPR001085), functioning primarily in the transfer of a methyl group from serine to tetrahydrofolate (THF), forming L-glycine and 5,10-methylenetetrahydrofolate (5,10-mTHF) [8]. This reaction is reversible and can form L-serine and THF from L-glycine and 5,10-mTHF as well. MTHFD2 and MTHFD2L are NAD(P)^+^-dependent methylenetetrahydrofolate dehydrogenase/cyclohydrolases (IPR000672), both mitochondrial. They catalyze the conversion of 5,10-methylenetetrahydrofolate to 5,10-methenyltetrahydrofolate (5,10-mTHF^+^) and 5,10-methenyltetrahydrofolate to 10-formyltetrahydrofolate (10-fTHF) [8]. These reactions, too, are reversible. Although frequently discussed as either NAD-dependent (MTHFD2) or NAD(P)^+^-dependent (MTHFD2L), both enzymes can utilize either cofactor, although their specificities and kinetics differ [17]. MTHFD1 (IPR000672 & IPR000559) is a trifunctional enzyme, capable of mTHF dehydrogenase, cyclohydrolase, and fTHF synthetase activities [8]. The various tetrahydrofolate intermediates in the process serve as C1 donors in the biosynthesis of nucleobases such as thymidine (from 5,10-mTHF) or purines (from 10-fTHF). In the mitochondria, MTHFD1L (IPR000672 & IPR000559) is a monofunctional formyltetrahydrofolate synthetase, despite having the same domain annotations as MTHFD1, and as the name suggests, can either convert 10-fTHF to formate and THF, or can synthesize 10-fTHF from formate and THF [8, 18]. While part of MTHFD1 is clearly homologous to MTHFD2 (IPR000672), MTHFD1 has an additional formyltetrahydrofolate synthetase, or FTHFS, domain (IPR000559). Non-vertebrate metazoans and nucletmyceans are known to have copies of *MTHFD1* and *MTHFD2*, but not *MTHFD1L* or *MTHFD2L*. Non-metazoans and non-nucletmyceans (i.e., non-opisthokonts) are known to have copies of *MTHFD2* (often called *FolD*), but not *MTHFD1*, *MTHFD1L,* or *MTHFD2L*. In these organisms, *FTHFS* is a standalone gene unlike in opisthokonts where it is fused with a methylenetetrahydrofolate dehydrogenase/cyclohydrolase domain (Fig. 1).

Its central role in primary metabolism and disease has made the folate cycle an investigative priority of the biomedical community for decades. In humans, abnormal folate cycle regulation has been implicated in a wide variety of diseases including various developmental neural tube defects, combined immunodeficiency and megaloblastic anemia, mitochondrial complex I deficiency, and oxidative cancers [11, 12, 19]. More generally, its role in growth and development make it important not only for human health, but for the health of agriculturally relevant animals and plants [20–22]. The folate cycle and its proper regulation are thus important not only for humans, but for systems that humans rely upon. However, despite the importance of this cycle to nearly every lineage across the tree of life, our knowledge of the folate cycle is confined largely to model systems (e.g. certain bacteria, mammals, plants, and yeasts) [15, 19–25]. Even so, these studies have revealed a rather interesting diversity of folate cycle enzymes. For example, it is apparent that MTHFD1L and MTHFD2L are specific to Vertebrata, as Saccharomyces do not possess a bifunctional dehydrogenase/cyclohydrolase as metazoans do, and certain enzymes in the folate cycle are even the products of ancient gene fusion events. Such a mosaic of folate cycle genes, whose diversity presumably expands beyond what has already been described due to us only having thoroughly searched a subset of RefSeq, make it a particularly interesting and important clade for studying processes of gene evolution. This combined with its relevance to most lineages makes the mTHF gene family one worthy of study not only for its own sake, but also due to its fundamental role in metabolism more broadly.

To address major gaps in knowledge pertaining to these aspects of the mTHF gene family, we conducted a wide-reaching effort to reconstruct the evolutionary history of the mTHF gene family across the Tree of Life. Our results indicate that the mTHF gene family has undergone repeated gene birth-and-death events, fusions with other folate cycle genes, and potential horizontal gene transfer events and/or instances of amino acid convergence across these groups, hence making it a highly dynamic gene family from an evolutionary perspective.

## Results

### Data acquisition

In total, we searched the coding sequences and protein sequences and/or genomes of 1288 species in RefSeq. The coding and protein sequences of 307 archaeans, 157 bacteria, 93 fungi, 186 invertebrates, 118 plants, 33 protozoans, and 364 vertebrates were analyzed (1123 species). We searched the genomes of an additional 165 species in RefSeq which either had no mTHF homologs in the coding/protein sets or which did not meet our criteria of having non-redundant GeneIDs for each sequence. These included 122 archaeans, 6 fungi, 5 invertebrates, 11 plants, 12 protozoans, and 9 vertebrates. Across these data sources, we identified mTHF homologs in 1137 species and were unable to identify homologs in the remaining 151 with the search criteria we used (SupplementaryData1/data/tables/summaries).

### Gene birth, death and convergence in Archaea

**Fig. 2:**
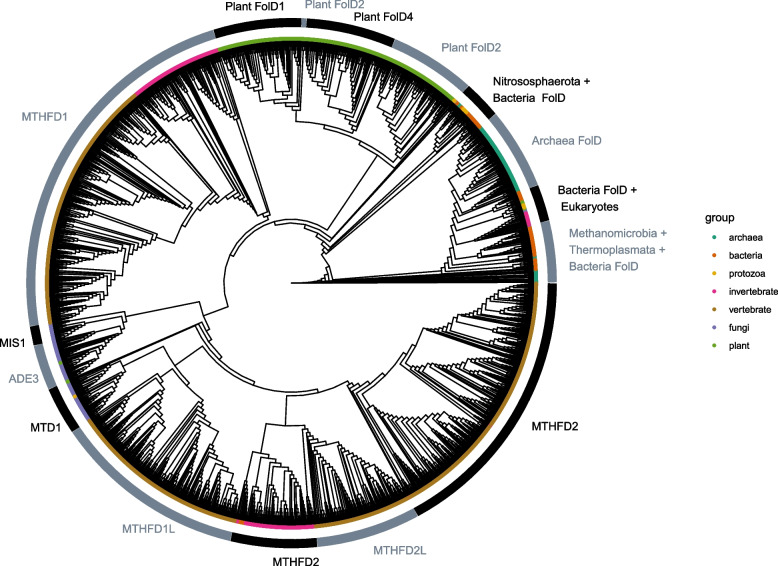
Phylogeny of the mTHF gene family. Circularized, unrooted maximum-likelihood nucleotide phylogeny of mTHF gene family members identified from across the Tree of Life. Tips were colored according to NCBI grouping and large-scale clades were annotated with bars to denote gene subfamily membership. Tip colors are assigned as follows: vertebrates = orange, invertebrates = purple, fungi = brown, protozoa = teal, plant = green, archaea = yellow, bacteria = black. Gene subfamilies were labeled with bars and subfamily names. Bar colors are only used to enhance contrast between subfamilies and do not necessarily denote relatedness. The topology suggests a history of several ancient and independent gene duplications events in plants (*FolD1*, *FolD2*, *FolD3*), fungi (*ADE3*, *MIS1*, *MTD1*), and vertebrates (*MTHFD1*, *MTHFD1L*, *MTHFD2*, *MTHFD2L*), as well as several recent gene duplication events that are not clearly visible at the scale of this phylogeny (see discussion on gene family evolution in Eukarya and SupplementaryData1/data/phylogenetics/). Relative to Eukarya, prokaryotes have experienced no ancient gene duplication events (i.e., in the stem groups of major phyla), although some lineages have experienced them more recently (see discussion on gene family evolution in Archaea and Bacteria and SupplementaryData1/data/phylogenetics/)

Analysis of gene presence and absence across Archaea reveal a number of lineages in which *FolD* is seemingly absent. At the ordinal level, assessed using the NCBI Taxonomy Browser, these include the Acidilobales, Archaeoglobales, Conexivisphaerales, Desulfurococcales, Fervidicoccales, Methanobacteriales, Methanocellales, Methanococcales, Methanopyrales, Nanobdellales, Sulfolobales, Thermococcales, and Thermofilales (SupplementaryData1/data/tables/results). The phylogeny of archean FolD sequences is incongruent with the current phylogenetic understanding of Archaea in that, in the former, archean sequences are polyphyletic whereas Archaea are usually understood as monophyletic or at least paraphyletic with the inclusion of eukaryotes. The majority of archean sequences do form a clade (Fig. 2, Archaea FolD), but Methanomicrobia, Thermoplasmata, and Nitrososphaerota sequences are nested within bacterial FolD sequences (Fig. 2, Nitrososphaerota+Bacteria FolD; Fig. 2, Methanomicrobia + Thermoplasmata + Bacteria FolD). This may be due to convergence upon certain amino acid motifs or horizontal gene transfer from bacterial species to archean species.

**Fig. 3:**
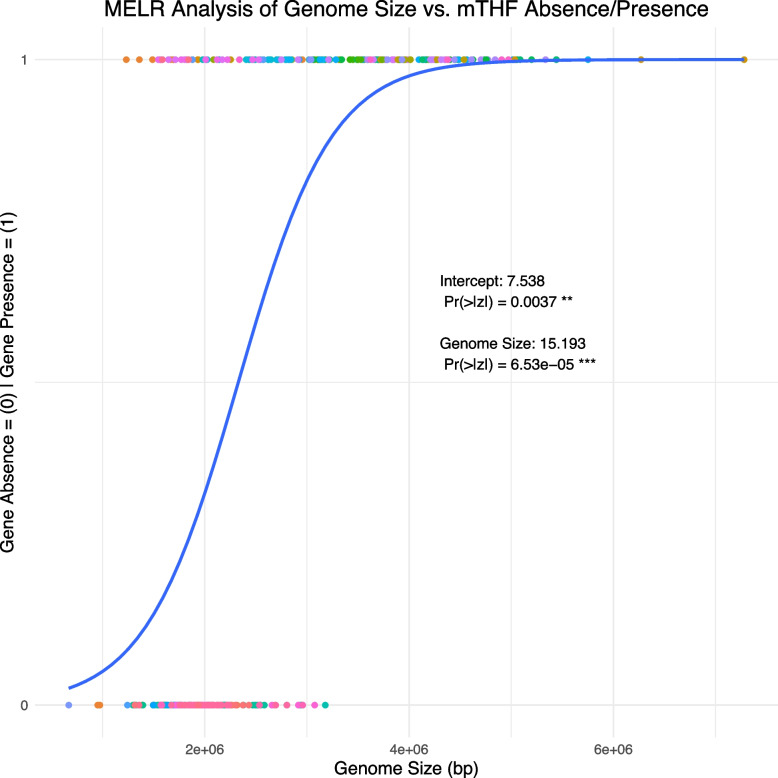
Mixed-effects logistic regression analysis of mTHF gene presence/absence in Archaea. Summary of mixed-effects logistic regression analysis of the relationship between genome size (in bp, x-axis) and mTHF gene absence and presence (0/1, y-axis) across 307 archean species. Genus membership was controlled for as a random effect. Dots represent individual species and dot color represents genus. Given that 122 genera were analyzed, genus-to-color relationships are not shown. Logistic sigmoid curve is seen in blue demonstrating that, in general, as genome size increases, the probability of mTHF gene family member presence increases (Genome Size = 15.193, Pr(>|z|) = 6.53e− 05)

Mixed-effect logistic regression analyses suggest there is a significant correlation between genome size and the absence/presence of *FolD* in archaean genomes (*p* = 6.53e− 05) (Fig. 3). Given that all genomes analyzed were complete assemblies, and given the thorough nature of our searches for mTHF homologs, we suggest this is likely a true relationship and not an artifact of genome completeness or the search parameters used. However, despite controlling partially for evolutionary distance between individuals, we could not control for it fully without a phylogeny.

### Dynamic evolution of eukaryotic FolD and FolD-like genes

The gene *FolD* encodes a bifunctional protein, capable of converting 5,10-methylenetetrahydrofolate (5,10-THF) to 5,10-methenyltetrahydrofolate (5,10-THF+) to 10-formyltetrahydrofolate (10-FTHF) [23]. This reaction is also reversible. *FolD* contains two domains, one that is capable of cyclohydrolase and dehydrogenase activities (PF00763.27, THF_DHG_CYH) and the other of which is capable of NAD(P)^+^ binding (PF02882.23, THF_DHG_CYH_C) (Fig. 1). Critical to the biosynthesis of thymidine and purines, *FolD* and its homologues are conserved across life, being found in nearly all Bacteria, Archaea, and Eukarya [15, 19, 23–25]. However, several lineages have also lost *FolD* entirely (SupplementaryData1/data/tables/results). Most bacteria and archaeans possess only a single copy of FolD, although this is not without exception. Notable expansions of this gene family are more widespread throughout eukaryotes. Indeed, land plants have three copies of *FolD*: *FolD1*, *FolD2*, and *FolD4*. *FolD3* is seemingly a pseudogene and is detectable in the genomes of some lineages such as the Brassicales (Fig. 2, Plant FolD1/FolD4/FolD2). There is also a small clade composed of several *Cryptomeria japonica* (cypress) and *Physcomitrella patens* (moss) copies of FolD sister to FolD1 (Fig. 2, Plant FolD1/FolD2). These are almost exclusively annotated as FolD2 or FolD2-like (with the exception of two *C. japonica* sequences annotated as FolD4) and likely represent independent duplication events of the FolD genes which may have converged upon certain amino acid motifs. Indeed, unlike most plants, *P. patens,* for example, has six copies of *FolD* with no indication that any are pseudogenes. It remains unclear what relevance this expanded *FolD* geneset has for the physiology of the *P. patens* and other plants with expanded suites of *FolD*.

Although it is difficult to pinpoint the exact timing and relevant lineages, there was a gene fusion event between one copy of *FolD* and another folate cycle gene, *FTHFS*, in a common ancestor of Opisthokonta [26] (Fig. 2, MTHFD1/MIS1/ADE3). Indeed, throughout life, *FolD* and *FTHFS* represent two distinct loci within the genome, each encoding for distinct enzymes. However, in Opisthokonts, there is no distinct *FTHFS* locus or enzyme, but rather a combined *FolD-FTHFS* locus and enzyme with *FolD* (200–400 amino acids) at the N-terminus and *FTHFS* (500–700 amino acids) at the C-terminus. This gene is typically referred to as *MTHFD1* (800–1200 amino acids in most taxa), especially in the metazoan literature, is often represented as a single-copy ortholog, and the encoded enzyme is trifunctional with methylenetetrahydrofolate dehydrogenase, cyclohydrolase, and formylsynthetase activity [8, 15, 26] (Fig. 1). Thus, the trifunctional enzyme product is capable of carrying out all steps of the folate cycle except for that carried out by serine hydroxymethyltransferase (SHMT). In Saccharomycetales, *MTHFD1* (also called *ADE3* in fungi) experienced a duplication event, giving rise to *MIS1* (Fig. 2, MIS1/ADE3). *ADE3*/*MTHFD1* encodes for a cytoplasmic copy of the trifunctional enzyme whereas *MIS1* encodes for a mitochondrial copy with the same function [27]. *MTHFD1* experienced an independent gene duplication event in Vertebrata, giving rise to *MTHFD1L*. Like MIS1, MTHFD1L is mitochondrial, but rather than being trifunctional, it is monofunctional with only methylenetetrahydrofolate formylsynthetase activity [18]. This is despite having an N-terminus region with clear homology to FolD, albeit one with significant divergence.

The majority of metazoans possess a single copy of *FolD* (also called *MTHFD2*). However, in a common ancestor of Vertebrata, a gene duplication event gave rise to *MTHFD2L*, which is similar to MTHFD2 at the sequence and structural levels and has been demonstrated in vivo and in vitro to carry out the same basic metabolic functions of interconverting 5,10-mTHF and 10-FTHF [17, 28]. However, their relative expressions differ by developmental stage and they do differ slightly in cofactor specificity and overall kinetics [17]. In most fungi, the most likely ortholog of *FolD and MTHFD2* is methylenetetrahydrofolate dehydrogenase (*MTD1*)*.* Relative to *FolD, MTHFD2*, and *MTHFD2L MTD1* contains a high proportion of mutations and has been shown in vitro to have only cyclohydrolase function [25, 27, 29]. Thus, MTD1 primarily converts 5,10-mTHF to 5,10-mTHF^+^ and vice-versa. As is also the case with MTHFD1L, the presumed relaxed selection experienced by MTD1 has made its exact phylogenetic placement relative to other mTHF gene family members difficult.

All of the major groups discussed thus far, both taxonomically and at the gene family/subfamily level, do not necessarily capture the entire history of the mTHF gene family’s evolution. Indeed, at a more granular level, there have been other gene duplication and gene loss events within families and even genera. There may also be additional independent gene fusion events and subtle neofunctionalization events that are yet uncharacterized due to a lack of functional validation and need for greater taxonomic sampling. For example, we detect an astounding 15 copies of mTHF gene family members in the poppy, *Papaver somniferum*. Several fish in the genera *Oncorhynchus*, *Salmo*, and *Salvelinus* have 6 copies, a deviation from the standard 4 in most vertebrates, with other taxa having more yet. Of course, these data, particularly in eukaryotes with complex genomes, must be interpreted carefully due to factors such as contamination and genome incompleteness having the ability to influence copy number. For example, it is highly unlikely that *Equus asinus* (donkey) and *Equus quagga* (zebra) have typical mTHF copy numbers for vertebrates while *Equus caballus* (domesticated horse) has zero. We also find suspicious the several plant, protozoan, fungal, and invertebrate sequences that fall out within bacteria, which may more likely be contamination rather than several independent instances of convergence.

## Discussion

### Patterns of mTHF gene family gain and loss in Archaea

In our reconstruction of the mTHF gene family phylogeny, within archaeans, Methanomicrobia, Thermoplasmata, and Nitrososphaerota were nested in predominantly bacterial clades. This may represent independent instances of convergence at the amino acid level upon certain amino acid motifs, or horizontal transfer from bacteria to these archaean lineages. In the latter case, though, due to our significant downsampling of Bacteria, it is difficult to assess which lineage(s) may have transferred their genetic material to these archean lineages. Horizontal gene transfer (HGT) is an important evolutionary mechanism whereby organisms incorporate transferred segments of foreign DNA into their genomes. The feasibility of such a transfer is dependent on several factors including mechanisms of transcription, metabolic differences between species, etc. [30]. HGT events have been identified between Archaea and Bacteria via mechanisms such as transformation and transduction [31]. In either case, donors and recipients of genetic material are likely to be found in the same general habitats [32]. Many cases of HGT between Archaea and Bacteria are largely unidirectional, as evidence by the substantial acquisition of genomic material from a methanogenic Bacteria ancestor by ancestral Haloarchaea, Archaeoglobales, and Thermoplasmatales [33].

Across Archaea, several groups also lack the key folate cycle gene *FolD* [23]. It is thus uncertain how the synthesis of key intermediates in nucleotide biosynthesis (e.g. 5,10-methylenetetrahydrofolate, 10-formyltetrahydrofolate) occurs. One possibility is that alternative pathways have evolved for the biosynthesis of said intermediates. It is not entirely uncommon for species to evolve alternative means of carrying out primary metabolic processes. It may also be possible that such compounds have been replaced by structurally similar analogues, or that the necessary compounds are scavenged from the environment [34]. Energy metabolism, an important set of processes in all domains of life, may be seen as an example of such metabolic adaptation. In aerobic environments, many eukaryotes ultimately rely on a combination of glycolysis, the tricarboxcylic acid cycle, and the pentose phosphate pathway for the regeneration of ATP from ADP and for the regeneration and biosynthesis of many important cofactors and non-cofactor metabolites. A dramatic contrast to this strategy can be seen in obligate anaerobes, many of which use fermentation or anaerobic respiration to regenerate energy currency, some of which may rely entirely on inorganic electron donors such as sulfates and nitrates rather than saccharides such as D-glucose. This can also be seen in certain Archaea that inhabit hypersaline ecosystems such as halophilic Archaea that have evolved a multitude of strategies allowing them to survive in such conditions [35]. In general, halophiles use amino acids and carbohydrates as carbon and energy source in contrast to many eukaryotes and prokaryotes which also depend heavily on lipids [36]. For example, *Halorhabdus utahensis* and *Haloterrigena turkmenica* use a non-oxidative cycle and transhydrogenase for NADPH regeneration instead of an oxidative pentose phosphate pathway [36].

There have also been many examples of non-homologous gene replacement in Archaea for carbohydrate metabolism. Archaea contain many enzymes needed for conversion that are not found in either Bacteria or Eukarya [37]. Metabolic processes have been found in central archaean metabolism that are highly conserved within Bacteria and Eukarya. These gaps are the products of the non-homologous gene replacement. The gene replacement products are seen in the Embden-Meyerhof-Parnas (EMP) pathway of halophilic Archaea, specifically in the fructose phosphorylation process. Fructokinase starts the process by phosphorylating fructose in fructose-1-phosphate (F1P) [38]. Archaea use ketohexokinase (KHK) instead of fructokinase to accomplish this [39]. As a result, it has been thought that halophiles contain a novel, nonhomologous enzyme for fructose phosphorylation. Many new enzyme families have been discovered in Archaea that are not seen in other domains of life such as FBP aldolases used in fructose 1,6-biphosphate cleavage [40].

Overall, Archaea have been generally thought to have a mosaic character. This is due to their overlap in bacterial and eukaryotic properties [39]. Nonetheless, Archaea have their own unique characteristics, which can be seen in their metabolism. There have been many examples of the evolution of new genes as well as modifications to pre-existing genes [41]. Though the folate cycle has not been thoroughly investigated within Archaea, it is plausible that we would see neofunctionalism and adaptation to the metabolic pathway, as was shown in carbohydrate metabolism. The diversity in metabolism and phylogenetics seen within Archaea that live in extreme environments highlight the possibility that this domain may be using alternative paths to process folate.

### mTHF gene family evolution is dynamic across Eukarya

The mTHF gene family, particularly in eukaryotes, is remarkably diverse in both the architectures of their constituents as well as in their functions. This has been raised in previous work that focused mostly on the evolution of the mTHF gene family in Opistokonta [10]. Here, we expand upon the current state of knowledge pertaining to the evolution of this gene family by rounding out the remainder of Eukarya, insofar as we are able given the availability of high-quality genomic resources.

It is probable that the most recent common ancestor of Eukarya had a suite of mTHF gene family members highly resembling that of archaeans and bacteria with one or two copies of *FolD*. This can be inferred not only from the data suggesting Eukarya likely diverged from Archaea over one billion years ago, but from the resemblance in mTHF gene family members between most archaean, bacterial, and eukaryotic lineages [42]. Indeed, it appears that several bikont protozoan, unikont protozoan, and plant lineages one or more copies of *FolD* and one or more copies of *FTHFS*, both of which are broadly similar in primary sequence structure to their archean *FolD* and *FTHFS* orthologs. However, it does appear that gene duplication events, primarily of *FolD*, have occurred throughout the Eukarya. In not all cases are these duplications necessarily synapomorphies of the larger group, many appear to be apomorphic. Of course, the degree to which these duplications are synapomorphies, symplesiomorphies, or apomorphies is highly dependent upon our current understanding of the evolution of these taxa, which for many such groups is still in a state of flux. It also depends greatly on the completeness of available data to assess gene presence, absence, possible duplication events and losses, etc., as well as the extent to which gene sets and genomes are contaminated with exogenous sequences.

However, in all Bikonta *FolD* and *FTHFS* are always found in their ancestral forms, as bidomain and unidomain genes respectively, encoding for enzymes of approximately 200–400 residues and 500–700 residues (Fig. 1). Previously, we suggested that in the Opisthokonta one of the two copies of ancestral *FolD* and *FTHFS* fused to form a chimeric gene, commonly referred to as *MTHFD1* [10]. The enzyme product of *MTHFD1* has the methylenetetrahydrofolate dehydrogenase and cyclohydrolase activities of FolD as well as the formyl-THF ligase activities of FTHFS, in one enzyme usually between 800 and 1200 resides in length. Here, we have provided additional evidence in support of this hypothesis. Assuming a fusion of *FolD* and *FTHFS* in a common ancestor of Opisthokonta gave rise to *MTHFD1*, it would be necessary for such an ancestor have two copies of *FolD* given that we see both *MTHFD1* and *FolD* in extant opisthokont genomes. In both Saccharomycetales and Vertebrata, we see independent duplications of *MTHFD1*, giving rise to one copy that is often expressed in the cytosol and one that is often expressed in the mitochondria. In Saccharomycetales, these copies are referred to as *ADE3* and *MIS1* respectively, both retaining full trifunctional abilities. In Vertebrata, these copies are referred to as MTHFD1 and MTHFD1L, with the latter having accumulated several deleterious mutations in the FolD region of the gene, thus rendering the enzyme product capable only of formyl-THF ligase activity. Thus, MTHFD1L is functionally equivalent to FTHFS, but structurally is clearly a paralogue of MTHFD1 given the detectable FolD and FTHFS regions. While we do not foreclose the idea that *FolD* and *FTHFS* have fused in other taxa, or *FolD* and other genes, we do not detect evidence of it in this study.

Also of note is the duplication of *MTHFD2* in Vertebrata giving rise to the paralogue *MTHFD2L*, which for all intents and purposes is identical in function the MTHFD2, albeit with altered substrate specificity, enzyme kinetics, and spatiotemporal expression patterns [17]. We also observe some puzzling losses of *FolD* in Nematoda, which given the high-quality genomic resources available for the phylum, we have assessed to be real and not an artifact of incomplete data. Indeed, without *FolD* the question of how Nematoda converts 5,10-methylenetetrahydrofolate to 5,10-methenyltetrahydrofolate to 10-formyltetrahydrofolate (and vice versa) must be raised. These organisms must either have a novel strategy of synthesizing these compounds, a novel strategy that circumvents the need to synthesize these compounds, or are acquiring them by other means (perhaps from hosts or endosymbionts). Thus, in a sense, we see a pattern of convergent evolution between some Archaea and Nematoda in that both have lost their copy of *FolD*, the Nematoda being the only known case of mTHF gene family member absence in eukaryotes.

## Conclusions

Our results indicate that the mTHF gene family, one of the main drivers of C1 metabolism, has undergone repeated gene birth-and-death events, subfunctionalization and neofunctionalization, fusions with other folate cycle genes, etc., making it a highly dynamic gene family from an evolutionary perspective. Taken together, we suggest that this gene family has the potential to serve as a useful model for studying a variety of processes in gene evolution, including but not limited to those listed here. Given its central role in the metabolism of many taxa, our results also suggest new, interesting areas of exploration into the evolution of one-carbon metabolism, particularly in those lineages lacking apparent folate cycle genes.

## Methods

### Computational resources

All analyses described herein were performed on the Roar Collab High Performance Computing Cluster at The Pennsylvania State University running Red Hat Enterprise Linux 8.9 (Ootpa) in the allocated scratch space. Several custom scripts were written for this work and can be found in “SupplementaryData1/computational_information/scripts”. Lists of package versions and conda environments can be found in “SupplementaryData1/computational_information/versions/”.

### Criteria for identifying target genomes

A custom script was used to download and combine the assembly summaries for all genomes in RefSeq such that the highest quality genomes could be obtained. For eukaryotes, all coding sequence, protein sequence, and whole genome data hereafter described must have met the following criteria to be considered for analysis, using the terminology from the assembly summary column headers: the refseq_category must be “representative genome” or “reference genome”, the version_status must be “latest”, the assembly_level must be “Complete genome” or “Chromosome”, the genome_rep[resentation] must be “Full”, and the assembly_type must be “haploid”. For Archaea and Bacteria, given the number of complete genomes in RefSeq, all of the above apply except assembly_level must be “Complete genome”.

Generally, we aimed to use only the latest representative or reference genomes due to their quality relative to older, non-representative and non-reference assemblies. We specifically excluded contig and scaffold level assemblies due to the increased likelihood (relative to complete and chromosome-level assemblies) of them being incomplete, which could lead to inaccurate inferences about gene content. We excluded genomes having “Partial” representation for the same underlying reason. We excluded all but haploid assemblies to reduce the risk of identifying gene duplicates erroneously. For all species except Bacteria, if a single species (including hybrids and strains) was represented by multiple assemblies, only one assembly was kept per species to reduce redundancy. For Bacteria, due to the large number of assemblies, if a single genus was represented by multiple assemblies, only one assembly was kept per genus. These criteria naturally excluded several “groups” such as metagenomes, plastids, plasmids, etc. We manually excluded viral data. All summary tables can be found in “SupplementaryData1/data/tables/summaries/”.

### Identifying mTHF homologs

The profile hidden Markov models (pHMMs) for the tetrahydrofolate dehydrogenase/cyclohydrolase, NAD(P)-binding domain (PF02882.23, THF_DHG_CYH_C) and the tetrahydrofolate dehydrogenase/cyclohydrolase, catalytic domain (PF00763.27, THF_DHG_CYH) were downloaded manually from InterPro and were concatenated into a single file. Each pHMM represents a probabilistic model of protein composition including site-wise variability based on alignments generated from sequences curated from across the Tree of Life. We opted to use HMMER (v3.1b2) for pHMM-based sequence homology searches due to higher sensitivity of this method to identify homologs relative to BLAST, especially remote homologs and pseudogenes [43]. This is due to the inherent sequence diversity captured in a single well-constructed pHMM query, which to achieve with BLAST would effectively require several separate queries.

For each genome, the coding (cds_from_genomic.fna.gz) and protein (protein.faa.gz) sequences were downloaded via rsync (v3.1.3). FASTA headers were renamed as “AssemblyAccession_cds/pro_SequenceAccession_GeneID” for simpler parsing. GeneID was included so that where multiple transcripts from a single gene were identified as hits, a single transcript from each gene could be chosen. Assemblies whose coding sequences were missing GeneIDs were excluded from further analysis as gene origin could not be established. All coding sequences were concatenated into a single database, as were all protein sequences.

The HMMER program hmmsearch was used to search the concatenated pHMMs protein sequence database with the following relevant parameters: -E 1e-10 --domE 1e-10 --incE 1e-10 --incdomE 1e-10. As two domains known to occur together were used in the search, most target sequences were expected to have multiple hits. Thus, a custom script was used to extract and deduplicate all target sequence accessions from the results files. These accessions were then used to gather an equivalent list of coding sequence accessions. One transcript was identified per GeneID, specifically the first occurance sorted alphanumerically. Each accession list was used to extract the relevant sequences from the downloaded coding sequence and protein sequence FASTA files. This was done such that protein and nucleotide sequence phylogenies could be reconstructed and topologies generally compared.

Genome assemblies (genomic.fna.gz) that were not properly formatted or which had no mTHF sequences identified via hmmsearch were then downloaded via rsync (v3.1.3). FASTA headers were renamed as “AssemblyAccession_gen_SequenceAccession” for simpler parsing. The HMMER program nhmmer was used to search against a *D. melanogaster* coding sequence query (NP_476929.1), chosen due to its high annotation quality, against each whole genome with the relevant parameter -E 1e-10 set. No sequences were extracted from the genome assemblies, but absence/presence was recorded. All hmmsearch and nhmmer results can be found in “SupplementaryData1/data/tables/results/”.

### Analysis of mTHF absence / presence

Using the hmmsearch and nhmmer results, a custom script was used to generate a table containing the following data: a 0 indicating a lack of identifiable mTHF gene family members or a 1 indicating the presence of one or more mTHF gene family members (i.e., one or more hmmsearch hits) (column 1), the number of mTHF copies found in the gene sets/genomes (column 2), the ungapped haploid genome size in bp (column 3), the genome accession (column 4), the group name (column 5), and the species name (column 6). We assessed absence and presence based on whether a species had either an hmmsearch or nhmmer hit (present) or neither an hmmsearch nor an nhmmer hit (absence). We also assessed copy number by identifying from the hmmsearch results how many unique hits were returned. We did not assess the copy number for genomes in which hits were found. This data can be found in “SupplementaryData1/data/tables/results/”.

### Analysis of mTHF gene family evolution in Archaea

Due to the generally small size and relative simplicity of their genomes, variability in genome sizes, the high proportion of complete and well-annotated genomes available in RefSeq, and the high rates of mTHF gene loss, we chose to examine the correlation between genome size (i.e., reduction) and mTHF gene family absence and presence (i.e., loss and gain or retention) in Archaea. We did not carry out these analyses for eukaryotes due to the lack of confidence in true, complete, ungapped genomes, genome size estimates, and the complexity of most eukaryotic genomes relative to Archaea.

All archaean entries were thus extracted from the aforementioned table and imported into R (v3.5.1). Entries from chromosome-level assemblies were excluded and accession and assembly level data were stripped, leaving only genus names, genome sizes, and binary mTHF absence (0) / mTHF presence (1) data. Genome sizes were standardized using the “scale” function and a mixed-effects logistic regression model was fitted using the glmer function from the R package lme4 (v1.1_17) with genus name as the random effect, standardized genome size as the fixed effect, and gene absence/presence as the dependent variable. This approach is more appropriate than a simple logistic regression analysis due to the confounding influence of phylogenetic relatedness on genome size and gene absence/presence [44]. However, this is an imperfect means of controlling for phylogenetic relatedness given that only genus-level relationships are controlled for. While phylogenetic logistic regression would be a better alternative, reconstructing a high-quality, multi-gene phylogeny of several hundred species of Archaea is far beyond the scope of this study. The R (v4.1.3) package ggplot2 (v3.4.2) was used to plot this data.

### Phylogenetic analysis of the mTHF gene family across Archaea and Eukarya

The extracted nucleotide and protein data hmmsearch results were separately aligned with MAFFT (v7.505) using the --auto setting. Alignments were then trimmed with trimAl (v1.4.rev15) using the -automated1 setting. Truncated sequences (consisting of at least 80% gap) were identified with the trimAl “get_sequences_gaps_ratio.py” Python script and removed from the alignment using the -selectseqs option. Sequences with 100% identity were then deduplicated with the rmdup script from the seqkit (v2.6.1) package. Best-fit models of evolution (SYM + G4 for coding) were identified and phylogenies reconstructed in IQ-Tree2 (v2.1.4-beta) by setting the following parameters: --seed {1–3} --fast -m TEST --merit BIC. IQ-Tree2 was run three times per dataset each with a different seed. IQ-Tree2 performed standard model searches, the best-fit models were selected via BIC, and the phylogenies were inferred. The resulting phylogenies were examined for long branch outliers (defined as branches 5 standard deviations above the mean branch length) and tip labels stemming from such branches common to all three topologies were used to remove sequences from the raw fasta files. IQ-Tree2 was run once more as above on the filtered dataset. Although attempts were made throughout this analysis to ensure the final nucleotide and protein datasets contained exactly the same sequences, this proved unfeasible, and we experienced significant sequence loss in our protein dataset using the aforementioned trimming parameters. As such, only the coding sequence dataset was used hereafter. The resulting maximum-likelihood newick files were imported into R (v4.1.3) and the following packages were loaded to annotate the phylogenies: ape (v5.7–1), dplyr (v1.1.2), ggplot2 (v3.4.2), ggtree (v3.2.1), tidytree (v0.4.4), and treeio (v1.18.1). The phylogenies were left unrooted and distinctive clades were labeled. Due to its scale, ultrafast bootstrap support values can be observed in the full figure in “SupplementaryData1/data/phylogenetics/.

## Data Availability

The raw data supporting the conclusions of this article are available in the National Center for Biotechnology Information GenBank and RefSeq FTP server (https://ftp.ncbi.nlm.nih.gov/genomes/). Sequence alignments, phylogenies in newick format, tables, and all other raw data not accessible via NCBI are available through Penn State ScholarSphere.  (https://scholarsphere.psu.edu/resources/2ee2a134-29c8-4c3c-8a8f-eaa28ed770d8).
